# Host Gene Expression of Macrophages in Response to Feline Coronavirus Infection

**DOI:** 10.3390/cells9061431

**Published:** 2020-06-09

**Authors:** Yvonne Drechsler, Elton J. R. Vasconcelos, Lisa M. Griggs, Pedro P. P. V. Diniz, Ellen Collisson

**Affiliations:** 1 College of Veterinary Medicine, Western University of Health Sciences, Pomona, CA 91766, USA; lgriggs@westernu.edu (L.M.G.); pdiniz@westernu.edu (P.P.P.V.D.); ecollisson@westernu.edu (E.C.); 2 Leeds Omics, University of Leeds, Leeds LS2 9JT, UK

**Keywords:** macrophages, transcriptome, gene expression, feline coronavirus, host response, innate immunity

## Abstract

Feline coronavirus is a highly contagious virus potentially resulting in feline infectious peritonitis (FIP), while the pathogenesis of FIP remains not well understood, particularly in the events leading to the disease. A predominant theory is that the pathogenic FIPV arises from a mutation, so that it could replicate not only in enterocytes of the intestines but also in monocytes, subsequently systemically transporting the virus. The immune status and genetics of affected cats certainly play an important role in the pathogenesis. Considering the importance of genetics and host immune responses in viral infections, the goal of this study was to elucidate host gene expression in macrophages using RNA sequencing. Macrophages from healthy male cats infected with FIPV 79-1146 ex vivo displayed a differential host gene expression. Despite the virus uptake, aligned viral reads did not increase from 2 to 17 h. The overlap of host gene expression among macrophages from different cats was limited, even though viral transcripts were detected in the cells. Interestingly, some of the downregulated genes in all macrophages were involved in immune signaling, while some upregulated genes common for all cats were found to be inhibiting immune activation. Our results highlight individual host responses playing an important role, consistent with the fact that few cats develop feline infectious peritonitis despite a common presence of enteric FCoV.

## 1. Introduction

Feline coronavirus (FCoV) is a highly contagious virus that is distributed worldwide and is ubiquitous in virtually all cat populations, particularly in multi-cat environments, such as shelters and rescues [1,2,3,4]. FCoV exists as two pathotypes, feline enteric coronavirus (FECV) and feline infectious peritonitis virus (FIPV). The enteric virus, FECV, commonly causes an asymptomatic infection presenting with mild gastrointestinal signs, and can persist in certain individuals [5]. FECV is readily transmitted via the fecal-oral route; therefore, the prevalence of FECV infection is generally associated with the number and density of cats housed together [1,4,6]. 

Sporadically and unpredictably, the infection can turn pathogenic, in which case the FIPV infection is associated with the highly fatal, systemic immune-mediated disease, feline infectious peritonitis (FIP) [5]. In this form, the virus develops a 100% fatal syndrome with two possible presentations: A pyogranulomatous disease of the visceral serosa and omentum, with consequent cavitary effusions, termed the “wet” or “effusive” form; and the dry form with granulomatous inflammation of parenchymatous organs such as kidneys, mesenteric lymph nodes, liver, pancreas, and central nervous system [7]. 

The pathogenesis of FIPV strains is still not fully understood, and several different theories are being discussed. A predominant theory has been that the pathogenic FIPV was caused by an FECV-like virus that had mutated such that it could acquire tropism for monocytes/macrophages. The implication was that FECV was present only in the intestines, while FIPV would now replicate mainly in monocytes, which subsequently transport the virus systemically, causing the disease. A study investigating strains FIPV 79-1146 and FECV 79-1183 comparing an ex vivo replication in cultured monocytes/macrophages of cats indicated a differential replication between the two viruses [8]. The authors speculated that a mutation potentially enables the FIPV to replicate more efficiently in monocytes and consequently, aids in spreading systemically in the cat. However, FECV has been shown to be present systemically in monocytes [9,10]. Pedersen et al. (2012) further showed that FIPV was present in feces of experimentally infected cats, but the virus was not infectious when tested via the oral-nasal route [11]. Pedersen inferred that intestinal and monocyte-macrophage tropisms of FECVs and FIPVs are related but not identical and that the mutational transformation of an FECV to FIPV might rather be happening in the monocytes/macrophages. These results show that analyzing responses of macrophages to the feline coronavirus is crucial for a better understanding of virus-host interactions.

A strong argument can also be made for the role of the immune status and genetics of affected cats in the pathogenesis. The latter theory is supported by the higher incidence of infection in very young or geriatric cats, purebreds, specifically Siamese and Burmese, and immune-compromised animals, such as those previously infected with the feline leukemia virus or feline immunodeficiency virus [4,12,13,14]. Several studies also have shown an important role for overall immune status and functioning cell-mediated immunity [15,16], antibody-mediated enhanced uptake of the virus [17,18], and numerous other factors such as destruction of lymphocytes in infected cats [19]. 

Another indication of the significance of the immune response in the pathogenesis is highlighted by the fact that inflammatory cytokine patterns play a role in the development of the dry or wet form of the disease [20,21,22]. However, little is known about the immunological pathways involved in early infection and the cells involved. The FIPV infection leads to a T-cell depletion in cats, although the virus is not directly infecting CD4^+^ or CD8^+^ T-cells [23,24]. The apoptosis of T-cells is probably caused by signaling mediators from other cells, such as infected macrophages or even epithelial cells [19,24]. 

Next-generation sequencing of RNA has increased our understanding of gene expression in response to pathogens in other viral infections and therefore, is a valuable tool to investigate early events in macrophage responses to the feline coronavirus. While more recent studies have investigated host responses to FIPV via transcriptome studies in Crandell-Rees feline kidney (CRFK) cells early after infection [25,26], peripheral blood mononuclear cells [26] or peritoneal cells including monocytes/macrophages [27] from infected cats, there are no reported transcriptome studies on primary monocytes/macrophages infected in the cell culture (ex vivo). In the current study, the transcriptome analysis of macrophages infected with FIPV 79-1146 was performed and analyzed for differential gene expression of ex vivo infected macrophages from six healthy male cats.

## 2. Materials and Methods

### 2.1. Cell Culture

CRFK cells (ATCC, Manassas, Virginia, cat#: CCL-94) were grown as monolayers in Dulbecco’s modified essential medium (DMEM) containing 10% of fetal bovine serum (FBS) and 1% of penicillin/streptomycin at 37 °C and 5% CO_2_.

### 2.2. Animal Procedures

All animal procedures were conducted and approved under the guidelines of the IACUC of Western University of Health Sciences, protocol approval number R10/IACUC/017. Peripheral blood for transcriptome studies was taken from six healthy male, specific pathogen-free (SPF) cats residing in an existing colony at the University of California, Davis. 30–40 mL of blood, equivalent to 1% of body weight or less was collected in heparinized tubes. The ages of cats at the time of blood draw were five months up to two years (five cats), and four years (one cat).

### 2.3. Monocyte Isolation

Monocytes from peripheral blood were isolated as previously described for canine monocytes [28] with some modifications. Briefly, the gradient centrifugation steps occurred at 450× *g* without break at deceleration and the subsequent washes to remove platelets were performed at 200× *g* for a total of three washes. PBMCs were counted, resuspended in RPMI 1640, containing 10% of FBS, 1% of penicillin and streptomycin, and 1× of non-essential amino acids, and plated at 5 × 10^6^ in 6-well plates. Non-adherent cells were removed after 24 h by vigorously washing with a culture medium and cells were infected the following day. 

### 2.4. Viral Infection for Transcriptome

For host transcriptome studies, macrophages were infected with FIPV 79-1146 (ATCC VR2128). The viruses were incubated at a multiplicity of infection (MOI) of 2 in a serum-free OptiMEM (Gibco, Thermo Fisher Scientific, Waltham, MA, USA) for 1 h for virus attachment, washed with OptiMEM, and incubated with fresh supplemented RPMI1640 for an additional 2 or 17 h. Technical replicates for the control, 2 and 17 h for macrophages from each cat were plated and incubated with PBS or the virus, respectively. CRFK cells (including technical replicates) were also infected as the control at an MOI of 1 in OptiMEM, followed by incubation in a supplemented DMEM. Uninfected controls underwent the same process with PBS without the virus. After incubation, the cell culture medium was completely removed and 600 μL of TRIzol (Invitrogen, Thermo Fisher Scientific, Waltham, MA, USA) was added to each well, followed by RNA extraction with the ZymoResearch RNA kit (ZymoResearch, Irvine, CA) according to the manufacturer’s instructions. The RNA quality was evaluated via the Bioanalyzer (Agilent, Santa Clara, CA) and sent (1 μg per sample) for mRNA sequencing to Novogene, Inc. (Sacramento, CA, USA). 

### 2.5. Quality Control of RNA Sequence Data

RNA paired-end sequencing quality control was assessed through FastQC (www.bioinformatics.babraham.ac.uk/projects/fastqc). An average of 35 million paired reads were sequenced per sample. Both adapters and low-quality bases (QV < 20) were trimmed from the reads’ extremities with Trimmomatic [29].

### 2.6. Alignment Against Reference Transcriptomes

Kallisto [30] was the algorithm of choice for performing the alignment of all paired reads against the whole *Felis catus* reference transcriptome (*F. catus* NCBI-RefSeq-9.0). An average of 87.5% of the total reads from each sample was mapped onto the cat’s annotated transcriptome. Alternatively, we also attempted to retrieve viral reads for both macrophages and CRFK from the sequenced libraries using Kallisto to align reads against the 11 protein-coding genes from the feline coronavirus (FCoV). An average of 0.03% of the total reads per sample was aligned against the FCoV annotated transcripts. Viral read counts were normalized as fragments per kilobase per million (FPKM): [read_counts / (gene_length_in_kb × total_reads_in_sample)] × 1,000,000.

### 2.7. Differential Expression

Tables generated by Kallisto were used as input for differential expression (DE) analyses. Due to the unique host responses in the macrophage dataset, the NOISeq version 2.14.1 [31] (Ctrl, 2, and 17 h) was employed to assess differentially expressed genes (DEGs) from each cat from which the ex vivo infected macrophages were derived. NOISeq output tables contained DEGs for each comparison (2 h versus control and 17 h vs. control) per cat. vennCounts and vennDiagram functions from the limma R package [32] were used for combining DEGs from each cat. Datasets were submitted to a multidimensional scaling (MDS) analysis, with the plotMDS function from EdgeR, to identify distinct samples clustered in a two dimensions-reduction landscape prior to the start of DE analyses. All tools described above for differential expression were run within the R environment version 3.5.2.

### 2.8. Gene Enrichment Analyses Using Both GO Terms and KEGG Pathways

After generating a list of differentially expressed genes (DEGs), we used ClueGO [33] under the Cytoscape version 3.7.1 [34,35] for a gene enrichment analysis relying on the *Felis catus* annotation from both gene ontology (http://geneontology.org/) and KEGG pathways (https://www.genome.jp/kegg/pathway.html) consortia. Both enrichment analyses adopted the Hypergeometric test along with the Benjamini and Hochberg *p*-value adjustment method. A 0.05 threshold was set for the latter.

## 3. Results

### 3.1. Host Responses Are Unique to Individual Cats with Several Clusters Present

**Figure 1:**
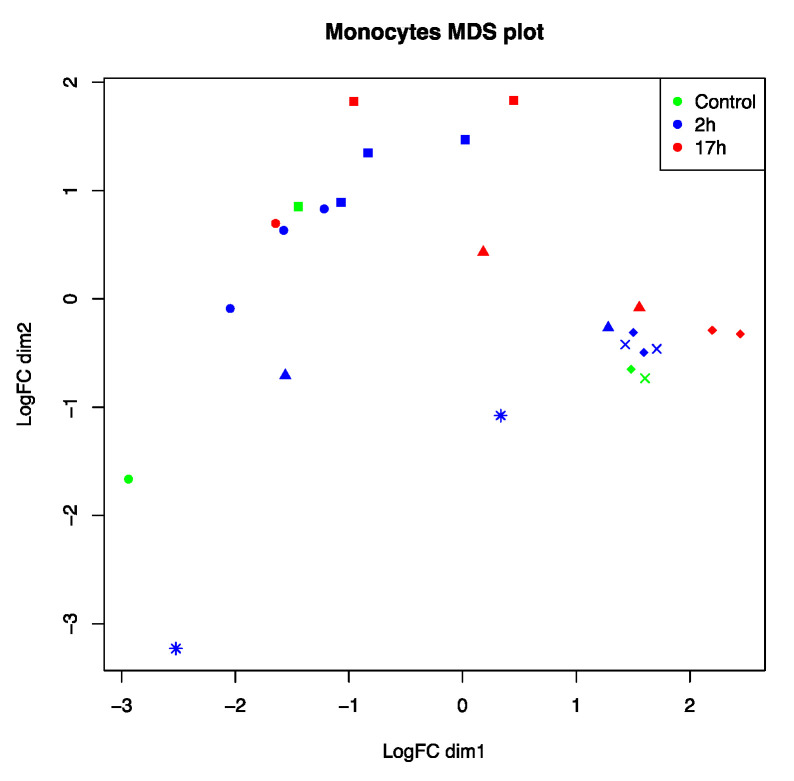
Multidimensional scaling (MDS) analysis on 27 RNA-seq samples from feline macrophages infected with feline infectious peritonitis virus (FIPV) at two different time points (2 and 17 h). Colors indicate infection status: Non-infected (green), 2 h of infection (blue), and 17 h of infection (red). Macrophages were derived from six different cats that are represented by distinct symbol shapes on the chart: Cat #1 (circle), cat #2 (square), cat #3 (asterisk), cat #4 (triangle), cat #5 (diamond), and cat #6 (cross symbol).

To analyze host responses to FIPV, mRNA from feline macrophages (*n* = 6), infected ex vivo, was isolated and processed for next-generation sequencing. A unique bioinformatic analysis of differentially expressed genes (NOISeq) was necessary to compare gene expression. Individual cats displayed very different patterns of gene activation, with macrophages from some cats showing a clear separation of uninfected control versus infected samples (#1 and 2), while macrophages from other cats (#5 and 6) did cluster closely together independent of infection status or infection time (Figure 1). 

### 3.2. Analysis of the Presence of Viral RNA in Infected Macrophages and CRFK

Cultured macrophages were infected with the FIPV 79-1146 for 2 and 17 h before RNA was collected for the expression analysis of host genes and viral reads. The viral presence was very low in the samples as indicated by the aligned reads for most macrophage samples at 2 h. However, the presence in most samples did indicate that viral particles were taken up by the cells (Table 1). No viral reads were detected in macrophages from cat #3.

Comparing 2 to 17 h samples, there was no indication of viral amplification despite viral uptake, as no increase of viral reads was observed in macrophages at the later time point. The viral reads at 17 h were either similar or lower than the number of reads at 2 h. Macrophages from one cat (cat #3) did not show any viral presence at 2 h. In contrast to the macrophages exposed to the virus, RNA sequencing showed several log-fold increases of viral isolates in the CRFK cells used as replication controls from 2 to 17 h (Table 1). This indicated that very few viral particles entering CRFK cells result in a significant amplification of the virus. This is also associated with pronounced cytopathic effects, which was observed in CRFK, but not macrophages. 

### 3.3. Host Transcriptome Analysis Shows Cat Specific Responses to FIPV

**Figure 2:**
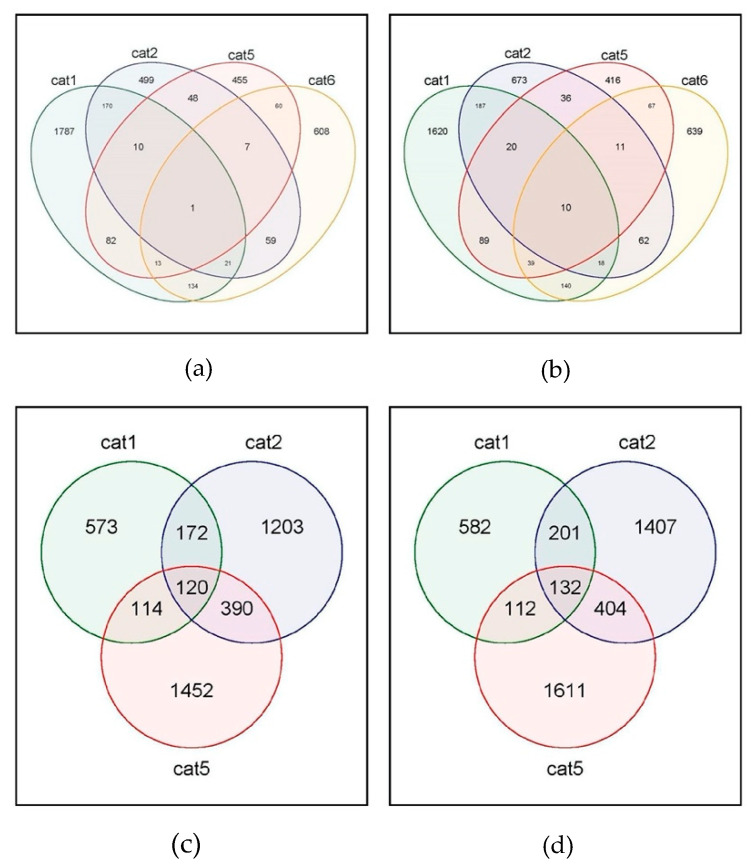
Venn diagram of differentially expressed genes in macrophages after infection with the feline coronavirus. (**a**) Number of genes downregulated at 2 h. (**b**) Number of genes upregulated at 2 h. (**c**) Number of genes downregulated at 17 h. (**d**) Number of genes upregulated at 17 h.

Macrophages from each of the individual cats did differentially express several hundred genes at 2 and 17 h, compared to uninfected macrophages, highlighting the unique host responses of the cells from individual cats. Cat #3 was excluded from the bioinformatic analysis due to the lack of viral RNA in the macrophages. Since no control sample was available for cat #4, no NOISeq individual analysis of 2 or 17 h samples of cat #4 was possible in comparison to its own uninfected control. At 2 h after infection, macrophages from cats #1, 2, 5, and 6 expressed 1787, 499, 455, and 608 differentially downregulated genes, while 1620, 673, 416, and 639 genes were differentially upregulated (Figure 2). 

It is of interest that, while the macrophages clearly show a differential regulation of genes, there are relatively few common DEGs expressed in all of the macrophages from the four cats that were compared. Only 10 genes were significantly upregulated in macrophages from all cats, while one gene was downregulated (Figure 2). The gene significantly downregulated in all macrophages was ATPase phospholipid transporting 8B4 (ATP8B4), which is involved in the cation transport and biosynthesis of ATP. Among the 10 upregulated genes, several are involved in immune signaling of the viral infection, such as SMAD4, ATP binding cassette, HAUS8, FCH domain, ubiquitin peptidase 20, and heteronuclear Ribonuclear Protein C (hnRNP C). The remaining genes in this group are involved in biosynthesis and other non-immune cellular functions (Table 2).

Monocyte isolations yielded different amounts of cells for each cat resulting in sufficient macrophages from only four cats to conduct the 17 h infection time point, with cat #3 not showing viral reads and thus not included in the analysis. Therefore, macrophage gene expression from cats #1, 2, and 5 was compared. At 17 h, macrophages from cats #1, 2, and 5 differentially downregulated 573, 1203, and 1452 genes, respectively, with 120 of the same genes downregulated in macrophages from all three cats (Figure 2). The number for upregulation of genes was similar, with 582, 1407, and 1611 genes for macrophages from cats #1, 2, and 5, respectively. One hundred and thirty-two genes were differentially upregulated in macrophages from all three cats (Figure 2). Downregulated immune genes at 17 h tumor necrosis factor (TNF) receptor superfamily 4, interleukin 10, transforming growth factor (TGF)-beta, signal transducer and transcription activator 3 (STAT3), and transcription factors interferon regulatory factor (IRF) 4, and SMAD family member 7 (Table A1). One hundred and thirty-four upregulated genes common for all cats included several other proteins involved in cell cycle and metabolism. Among these were several in the centromere or centrosomal category, nuclear body protein SP140, or 7-dehydrocholesterol reductase. There were only two classical cytokine genes among the upregulated, namely leukemia inhibitory factor (LIF), from the interleukin 6 cytokine family, and the transcription factor IRF 3 (Table A2). 

### 3.4. Gene Enrichment for Pathway Analysis and Ontology

As indicated in Figure 2, at 2 h after infection, there was no significant overlap of DEGs common to all infected macrophages in order to allow gene enrichment analyses (both GO terms and KEGG pathways). There was more commonality of DEGs expressed in response to FIPV at 17 h when macrophage responses of individual cats were compared, therefore gene enrichment was performed. Overall, 120 genes were differentially downregulated in all cats in response to FIPV with one KEGG pathway enriched, “Arginine and proline metabolism” (Table 3). 

Gene ontology for enriched genes downregulated at 17 h showed the terms “extracellular matrix binding” and “collagen binding” for the molecular function, and “regulation of endothelial cell proliferation”, “regulation of epithelial cell proliferation”, “positive regulation of vasculature development”, “positive regulation of angiogenesis”, and “regulation of peptidase activity” for biological processes (Table 4). 

KEGG pathways for enriched genes upregulated at 17 h were “Steroid biosynthesis”, “Valine, leucine and isoleucine degradation”, “Butanoate metabolism”, “Terpenoid backbone biosynthesis”, “Cell cycle”, “p53 signaling pathway”, and “Progesterone-mediated oocyte maturation” (Table 3). Gene ontology for this same set of DEGs resulted in terms related to several nuclear and mitotic cellular processes, including cytoskeletal and spindle organization (Table 4). 

**Figure 3:**
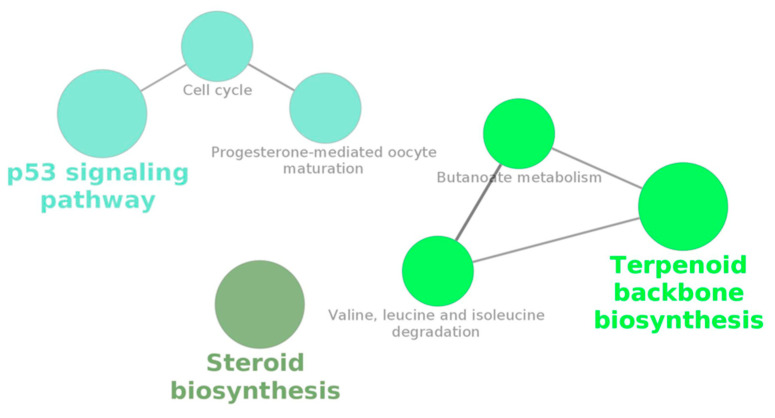
Cytoscape network output from a KEGG-based gene enrichment analysis performed by clueGO on the 132 differentially upregulated genes (main intersection from Figure 2d) in macrophages infected with the feline coronavirus (FCoV) for 17 h.

The Cytoscape network analysis (by clueGO) shows how several of these pathways interact with each other, yielding two networks (Figure 3).

## 4. Discussion

While isolates of both FCoV serotypes I and II have been shown to be pathogenic, only serotype II has been shown to efficiently replicate in the cell culture, using CRFK or *felis catus* whole fetus (FCWF) cell lines. Therefore, serotype II strains have been the subject of expanded in vitro and ex vivo investigations, including those focusing on viral entry and replication in monocytes/macrophages. It remains to be satisfactorily explained how both of these serotypes can arise from FECV in individual cats, with a yet unidentified mutation potentially resulting in a differential replication in monocytes and subsequently, leading to de novo pathogenesis in the affected cats. It is, however, likely that host immune responses play a significant, but not sufficiently elucidated, role in the pathogenesis, and the question remains: What are the responses by the macrophages when infected with FCoV?

The presence of the virus has been evaluated directly in studies in macrophages [8,36,37] using PCR or immunofluorescence. Most studies showing an increased replication of FIPV in vitro, particularly in comparison to FECV, use indirect methods such as TCID50 in CRFK cells incubated with virus-infected macrophage extracts or supernatants [8,37]. However, to our knowledge, no studies so far have verified replication in macrophages by more state-of-the-art sensitive techniques, such as RNA sequencing. 

In this study, RNA sequencing and analysis of viral reads indicated that at 2 h, the virus is taken up by the macrophages, as evidenced by the presence of viral RNA. However, there was no significant increase in viral RNA at 17 h, a time close to peak replication of the virus, which is usually at 24 h. Instead of a significant increase in viral reads, which would be expected with viral amplification, a similar amount, or even decrease of the virus was observed in the samples. In contrast to this, just a limited uptake of the virus into CRFK cells at 2 h led to several log-fold of replication, indicated by several thousands to hundreds of thousands of viral transcripts at 17 h. While the samples were not enriched for the pathogen, but rather the host RNA, there is a striking difference between replication of the virus in the macrophages compared to CRFK cells. Since subtle differences of replication in macrophages might not be recognized and could be below the threshold of detection, further investigations are warranted.

Macrophages of one cat #3 did not show the presence of viral RNA at all. This might be a technical issue, such as a higher MOI needed for successful infection. It is also possible that this particular host was more resistant to infection, which has been shown before both in vivo and in vitro [38,39]. Macrophages are competent immune cells and it has been shown that in vitro or ex vivo infection of macrophages with other viruses is challenging even if viremia exists in vivo. For example, there is evidence that Marek’s disease virus, a herpesvirus, is phagocytized by macrophages in vivo and then disseminated to infect T and B lymphocytes. In contrast, in vitro/ex vivo infection of macrophages has been shown to be difficult [40]. The investigators were successful when virally infected target cells were incubated with the macrophages, which then phagocytized the infected cells and therefore, took up the virus much more effectively. Similarly, it might be worth exploring the incubation of feline macrophages with infected epithelial target cells for studies requiring a higher ratio of macrophages positive for the viral antigen. In addition, methods such as RNA sequencing could be explored to better quantify differences between FIPV and FECV uptake, viral presence, and amplification in macrophages.

Gene expression of monocytes/macrophages following exposure to the feline coronavirus so far has not been investigated with next-generation sequencing. The PCR analysis of mRNA expression of individual cytokines and other immune-related proteins has been done in vitro and in vivo [19,20,21,41], but next-generation sequencing is poised to give a more complete picture of gene expression after infection. In particular, differences in responses to FIPV and FECV can be better defined. 

Transcriptome analyses of FIPV-infected feline macrophages were done in this study at 2 h (early phase of infection) and 17 h (closer to the known peak of amplification of FCoV), and samples were positive for a limited presence of viral transcripts. However, no enrichment of pathways or gene ontology common for all cats was possible in the early phase after infection. The only gene downregulated in all analyzed samples at 2 h was ATPase phospholipid transporting 8B4 (ATP8B4). This protein is involved in phospholipid transport, but there are no studies to its involvement in viral infection and replication. From the 10 upregulated genes, several are involved in immune-related host responses (Table A1). SMAD4 is a transcription factor involved in immune signaling, particularly in TGF-beta signaling. TGF-beta is an anti-inflammatory or regulatory cytokine that has been demonstrated to act as a proviral factor in epithelial cells during an influenza infection [42]. Haus8 is involved in maintaining cellular spindle integrity, but appears to have a role in the RIG-1 like antiviral signaling pathway in a Sendai virus infection [43]. ATP binding cassette subfamily A member 1, alternatively named RNase L inhibitor, blocks ribonuclease L. The interferon-regulated 2-5A/RNase L pathway plays a major role in the antiviral innate immune response. Consequently, several viruses are known to inhibit this pathway, including HIV which also induces the RNase L inhibitor [44]. The FCH domain is a phosphoprotein, associated with a viral infection. It is linked to endocytosis in an avian influenza virus replication [45]. Heterogeneous nuclear ribonucleoprotein C associated with pre-mRNA processing, RNA metabolism, and transport is a host factor important in the replication of positive-strand RNA viruses [46,47]. Finally, ubiquitin-specific peptidase 20 is an enzyme that has been shown to negatively regulate NFkB in an HTLV infection [48]. Other genes in this group are involved in cellular processes not linked to viral replication. Taken together, we identified several genes that are downregulated in all macrophages infected with FIPV and are linked to antiviral signaling. However, further studies are needed to elucidate the role of these genes in viral host interactions. 

Interestingly, several downregulated genes that did overlap among cats were involved in immune signaling, which might confirm that the immune responses of macrophages are negatively altered by FIPV. While a few of these genes are easily identifiable for involvement in antiviral or inflammatory responses, such as interferon regulatory factor 4 (IRF 4) or TGF beta, a further investigation shows that several of the transcripts do, in fact, play a role in responses to infection. For example, heterogeneous nuclear ribonucleoprotein A2/B1 (hNRP A2/B1), which is involved in RNA binding and trafficking, has previously been shown to bind to the NP of Avian Influenza [46]. Other proteins downregulated are purinergic receptors (P2Y), that are involved in antiviral responses, affecting cytokine responses and T-cell activation [49]. These receptors also contribute to the direct elimination of the virus by inhibiting their intracellular replication [50]. Intersectin 2 plays important role in the regulation of the adaptive immune response in viral infection [51]. Ubiquitin-like proteins have been shown to modify proteins, thus conferring functions related to programmed cell death, autophagy and regulation of the immune system [52], and IRF 4, which is critical to T-cell effector function [53]. 

In regards to the viral infection of macrophages, it would be expected to see a strong inflammatory response of the cell in response to the virus, such as upregulation of Toll-like receptor pathways, interferon type I signaling, etc. In our study, the absence of typical inflammatory signals is notable. While immune molecules IRF3 and LIF were upregulated, no classical virally activated signaling is discernable or enriched in KEGG pathways. LIF has not been well investigated in regards to viral infections, but it has been implicated in suppressing the replication of HIV [54]. IRF3 is necessary for IFN beta induction and SARS-CoV has been shown to block a step between the nuclear transport of IRF3 and its phosphorylation, which is necessary for an IFN induction, but not with the induction of IRF3 itself [55]. Our data is consistent with FCoV employing a similar strategy, however, further studies are needed.

Some differentially upregulated genes common for all cats, after a macrophage infection, were found to be within unexpected cellular functions, such as centromere proteins and TPX2 that are involved in specific phases of the cell cycle. Recent studies have indicated that centrosomes, as well as spindle organization, are actually involved in responses to a viral infection [56,57,58]. On the other hand, some upregulated transcripts included the protein tyrosine phosphatase, which is involved in attenuating T-cell activation [59] or nuclear body protein SP140. The latter has been shown to act as important repressor of genes involved in the regulation of cytokine production, inflammatory response, and cell-cell adhesion [60]. 

The picture emerging from these differentially expressed genes is that genes involved in antiviral responses and immune activation are depressed by FIPV uptake, while other genes involved in cell cycle and proteins repressing immune responses are upregulated. Even without an active viral replication, these changes most likely influence the pathogenesis and might explain how the monocytes carrying the virus potentially affect other cells, specifically lymphocytes. 

## 5. Conclusions

The FIPV exposure and uptake leads to a limited differential gene expression in feline macrophages that might affect cell function. It will be of importance to further investigate cellular responses to FCoV isolates, in order to understand virus interactions with the host macrophages. A comparison between infection with FIPV and FECV strains focusing on host responses might yield insights into the pathogenesis of the virus. On the other hand, individual cat responses may be found to be of significant relevance to the pathogenesis and the number of samples required for analyses might be very high. The problem remains that very few cells take up the virus, and thus it will be difficult to analyze the transcriptome of a low number of infected cells. However, with high sample numbers, it could still be possible to identify differential gene expression that can provide valuable insight into the pathogenesis. An alternative is to use bone marrow-derived macrophages for viral infection since these macrophages have been shown to support replication at a higher rate, especially at a high MOI. An attempt to increase the number of virus-positive cells by incubating them with infected target cells may also identify more infected macrophages. Another possible source of infected macrophages is to isolate and analyze cells directly from viremic cats for transcriptome studies, however, immunologically those cells are representative of an ongoing disease process, and gene expression probably would be very different than early in the pathogenesis. In any case, further analyzing the host responses with next-generation molecular techniques will increase our understanding of FIPV pathogenesis.

## Figures and Tables

**Table 1:** Viral RNA load per sample in both raw read count summation and normalized fragments per kilobase million (FPKM) average from all virus’ genes combined, obtained in cellular extracts of either macrophages or CRFK cells infected with FIPV, at 2 and 17 h post-infection. Cats #1–6 were healthy cats from the UC Davis cat colony, ages five months (5M) to four years (4Y). In macrophages, there was a limited viral presence/uptake of FIPV at 2 h, or not detected (nd), and no evidence of significant replication at 17 h (cat #6 n/a (no sample available)). In CRFK cells, viral RNA increased several log-fold from 2 to 17 h.

MØ Origin	Read Counts	Read Counts	FPKM Average	FPKM Average
(age of cat)	2 h	17 h	2 h	17 h
cat #1 (2Y)	727	2	4.46 × 10^−2^	1.38 × 10^−4^
cat #2 (2Y)	375	nd	1.82 × 10^−2^	nd
cat #3 (4Y)	nd	nd	nd	nd
cat #4 (5M)	1798	1555	9.93 × 10^−2^	9.05 × 10^−2^
cat #5 (2Y)	673	202	3.62 × 10^−2^	8.69 × 10^−3^
cat #6 (2Y)	245	n/a	1.4 × 10^−1^	n/a
CRFK 1	3715	459,899	1.38 × 10^−1^	17.4
CRFK 2	3	4871	2.19 × 10^−4^	1.54 × 10^−1^
CRFK 3	808	45,208	4.71 × 10^−2^	1.70
CRFK 4	1576	71,746	9.04 × 10^−2^	2.91

**Table 2:** Genes #1–10 were upregulated at 2 h in all samples, gene #11 was downregulated at 2 h in all samples.

#	Gene Identifier	Gene Name
1	XM_019815278.2	*SMAD family member 4, transcript variant X3*
2	XM_019816257.2	*ATP binding cassette subfamily A member 1, transcript variant X2*
3	XM_019819160.2	*HAUS augmin-like complex subunit 8, transcript variant X1*
4	XM_019819460.2	*FCH domain only 1, transcript variant X4*
5	XM_019832772.2	*heterogeneous nuclear ribonucleoprotein C (C1/C2), transcript variant X1*
6	XM_023240709.1	*pantothenate kinase 1, transcript variant X2*
7	XM_023240796.1	*ligand-dependent corepressor, transcript variant X4*
8	XM_023242837.1	*ubiquitin specific peptidase 20, transcript variant X4*
9	XM_023248013.1	*RAB interacting factor, transcript variant X2*
10	XM_023255193.1	*transcription factor 7, transcript variant X5*
11	XM_019832447.2	*ATPase phospholipid transporting 8B4 (putative), transcript variant X2*

**Table 3:** Enriched KEGG pathways of differentially expressed genes (DEGs) in macrophages 17 h after infection with FIPV. Term P-Value corrected: Corrected with Benjamini and Hochberg.

**KEGG Pathways 17 h Downregulated**					
KEGG ID	KEGG Pathway	Term *p*-Value	Term *p*-Value Corrected	Fold Enrichment	Associated Genes Found
KO 0000330	Arginine and proline metabolism	6.73 × 10^−4^	6.73 × 10^−4^	5.88	[CNDP2, OAT, ODC1]
**KEGG Pathways 17 h Upregulated**					
KEGG ID	KEGG Pathway	Term *p*-Value	Term *p*-Value Corrected	Fold Enrichment	Associated Genes Found
KO 0000100	Steroid biosynthesis	4.13 × 10^−6^	2.89 × 10^−5^	18.2	[DHCR7, FDFT1, LOC101083499, LSS]
KO 0000280	Valine, leucine, and isoleucine degradation	2.35 × 10^−3^	2.35 × 10^−3^	5.77	[AACS, ACAT2, HMGCS1]
KO 0000650	Butanoate metabolism	3.40 × 10^−4^	5.94 × 10^−4^	11.1	[AACS, ACAT2, HMGCS1]
KO 0000900	Terpenoid backbone biosynthesis	1.58 × 10^−4^	3.68 × 10^−4^	14.3	[ACAT2, HMGCS1, MVD]
KO 0004110	Cell cycle	3.89 × 10^−4^	5.45 × 10^−4^	4.07	[CCNB1, CCNB2, CHEK2, PLK1, TTK]
KO 0004115	p53 signaling pathway	3.27 × 10^−5^	1.14 × 10^−4^	6.85	[APAF1, CCNB1, CCNB2, CHEK2, GTSE1]
KO 0004914	Progesterone-mediated oocyte maturation	1.08 × 10^−3^	1.25 × 10^−3^	4.49	[AURKA, CCNB1, CCNB2, PLK1]

**Table 4:** Gene ontology enrichment for differentially expressed genes in macrophages 17 h after infection with FIPV.

**Gene Ontology for Genes Differentially Downregulated at 17 h**			
**GO Molecular Function Complete**	**Fold Enrichment**	**Raw *p*-Value**	**FDR**
extracellular matrix binding (GO:0050840)	90.54	6.35 × 10^−6^	2.37 × 10^−2^
collagen binding (GO:0005518)	61.83	1.85 × 10^−5^	3.45 × 10^−2^
**GO Biological Process Complete**	**Fold Enrichment**	**Raw *p*-Value**	**FDR**
regulation of endothelial cell proliferation (GO:0001936)	56.33	3.29 × 10^−8^	4.49 × 10^−4^
positive regulation of angiogenesis (GO:0045766)	34.14	6.07 × 10^−6^	2.76 × 10^−2^
positive regulation of vasculature development (GO:1904018)	31.01	8.77 × 10^−6^	2.99 × 10^−2^
regulation of epithelial cell proliferation (GO:0050678)	19.84	4.81 × 10^−6^	3.28 × 10^−2^
regulation of peptidase activity (GO:0052547)	16.31	1.22 × 10^−5^	3.33 × 10^−2^
**Gene Ontology for Genes Differentially Upregulated at 17 h**			
**GO Molecular Function Complete**	**Fold Enrichment**	**Raw *p*-Value**	**FDR**
condensed nuclear chromosome outer kinetochore (GO:0000942)	>100	1.99 × 10^−5^	3.32 × 10^−2^
**GO Molecular Function Complete**	**Fold Enrichment**	**Raw *p*-Value**	**FDR**
regulation of spindle organization (GO:0090224)	>100	4.63 × 10^−6^	1.26 × 10^−2^
establishment of spindle orientation (GO:0051294)	>100	4.63 × 10^−6^	1.05 × 10^−2^
establishment of spindle localization (GO:0051293)	83.29	8.53 × 10^−6^	1.16 × 10^−2^
spindle localization (GO:0051653)	69.41	1.41 × 10^−5^	1.75 × 10^−2^
mitotic spindle organization (GO:0007052)	48.43	3.88 × 10^−5^	3.79 × 10^−2^
mitotic sister chromatid segregation (GO:0000070)	45.52	2.18 × 10^−6^	1.49 × 10^−2^
microtubule cytoskeleton organization involved in mitosis (GO:1902850)	45.52	2.18 × 10^−6^	9.94 × 10^−3^
mitotic nuclear division (GO:0140014)	41.32	1.61 × 10^−7^	2.20 × 10^−3^
sister chromatid segregation (GO:0000819)	35.6	5.55 × 10^−6^	9.47 × 10^−3^
spindle organization (GO:0007051)	35.14	5.82 × 10^−6^	8.83 × 10^−3^
nuclear chromosome segregation (GO:0098813)	22.04	3.44 × 10^−5^	3.91 × 10^−2^
nuclear division (GO:0000280)	21.69	3.46 × 10^−6^	1.18 × 10^−2^
organelle fission (GO:0048285)	19.83	5.29 × 10^−6^	1.03 × 10^−2^
mitotic cell cycle process (GO:1903047)	13.3	3.49 × 10^−5^	3.66 × 10^−2^
microtubule cytoskeleton organization (GO:0000226)	12.48	4.69 × 10^−5^	4.27 × 10^−2^

## Appendix A

**Table A1:** Genes differentially downregulated in all macrophages 17 h after infection with FIPV.

Gene Identifier	Gene Name
NM_001009200.1	*TNF receptor superfamily member 4*
NM_001009209.1	*interleukin 10*
NM_001009253.1	*CD80 molecule*
XM_003980782.4	*transforming growth factor beta induced*
XM_003981485.5	*CD74 molecule, transcript variant X1*
XM_003981616.5	*growth arrest and DNA damage inducible beta*
XM_003984354.4	*limb bud and heart development, transcript variant X1*
XM_003986416.5	*PR/SET domain 1, transcript variant X1*
XM_003987780.4	*serine and arginine rich splicing factor 5, transcript variant X2*
XM_003987833.5	*NPC intracellular cholesterol transporter 2*
XM_003990035.4	*peroxiredoxin 1*
XM_003990688.5	*glycophorin C (Gerbich blood group)*
XM_003994088.5	*ribosomal protein S24, transcript variant X6*
XM_003995273.5	*carnosine dipeptidase 2, transcript variant X1*
XM_003996781.5	*post-GPI attachment to proteins 3, transcript variant X1*
XM_003998055.5	*metallothionein-1*
XM_003999680.5	*T-cell surface glycoprotein CD1b*
XM_006928964.4	*bromodomain and PHD finger containing 1, transcript variant X9*
XM_006929396.3	*transmembrane protein 140, transcript variant X2*
XM_006929637.3	*ribonucleic acid export 1, transcript variant X1*
XM_006929792.2	*AAR2 splicing factor homolog, transcript variant X1*
XM_006930307.4	*BCL2-like 11, transcript variant X1*
XM_006930765.3	*cathepsin B, transcript variant X2*
XM_006931414.4	*nucleoporin 153, transcript variant X3*
XM_006931511.2	*interferon regulatory factor 4, transcript variant X3*
XM_006932723.3	*mitochondrial ribosomal protein L52, transcript variant X5*
XM_006932762.3	*ring finger protein 31, transcript variant X2*
XM_006933673.2	*microspherule protein 1, transcript variant X4*
XM_006934848.4	*Rab geranylgeranyltransferase beta subunit, transcript variant X2*
XM_006934929.4	*coagulation factor III, tissue factor*
XM_006935700.4	*diacylglycerol kinase delta, transcript variant X2*
XM_006938342.3	*ankyrin repeat and LEM domain containing 2, transcript variant X2*
XM_006938804.4	*elastin microfibril interfacer 2, transcript variant X1*
XM_006940068.4	*zinc finger protein 207, transcript variant X5*
XM_006941006.4	*CD37 molecule, transcript variant X5*
XM_006943029.3	*Rho GTPase activating protein 30, transcript variant X2*
XM_011282426.3	*ELOVL fatty acid elongase 5, transcript variant X3*
XM_011282594.3	*BCL2 associated transcription factor 1, transcript variant X3*
XM_011283077.3	*thrombospondin 1*
XM_011283790.3	*C-type lectin domain family 4 member A, transcript variant X2*
XM_011285121.3	*extracellular matrix protein 1, transcript variant X4*
XM_011285878.3	*ATPase 13A3, transcript variant X1*
XM_011286809.3	*CD44 molecule (Indian blood group), transcript variant X13*
XM_011290903.3	*coagulation factor V*
XM_011291023.2	*quinone oxidoreductase-like protein 2, transcript variant X1*
XM_011291505.3	*poly(U) binding splicing factor 60, transcript variant X5*
XM_011291709.2	*RNA binding motif protein 3, transcript variant X1*
XM_019811387.2	*hypoxia upregulated 1, transcript variant X5*
XM_019811602.2	*family with sequence similarity 76 member B, transcript variant X1*
XM_019812261.2	*CD44 molecule (Indian blood group), transcript variant X2*
XM_019812263.2	*CD44 molecule (Indian blood group), transcript variant X3*
XM_019812267.2	*CD44 molecule (Indian blood group), transcript variant X7*
XM_019812269.2	*CD44 molecule (Indian blood group), transcript variant X10*
XM_019812271.2	*CD44 molecule (Indian blood group), transcript variant X12*
XM_019815319.2	*cold inducible RNA binding protein, transcript variant X2*
XM_019816708.2	*TSC complex subunit 1, transcript variant X3*
XM_019817540.2	*HEAT repeat containing 6, transcript variant X4*
XM_019817905.2	*signal transducer and activator of transcription 3, transcript variant X2*
XM_019819573.1	*heterogeneous nuclear ribonucleoprotein L, transcript variant X3*
XM_019819600.2	*ubiquitin-like modifier activating enzyme 2, transcript variant X2*
XM_019820314.1	*Sad1 and UNC84 domain containing 1, transcript variant X5*
XM_019821314.2	*signaling lymphocytic activation molecule family member 1, transcript variant X2*
XM_019821551.2	*cellular repressor of E1A stimulated genes 1, transcript variant X2*
XM_019822490.2	*ubiquitin associated protein 2-like, transcript variant X12*
XM_019822721.2	*YTH N6-methyladenosine RNA binding protein 3, transcript variant X5*
XM_019823162.2	*Src-like adaptor, transcript variant X1*
XM_019823774.1	*RNA binding motif protein 3, transcript variant X2*
XM_019823775.1	*RNA binding motif protein 3, transcript variant X4*
XM_019824901.1	*ubiquitin specific peptidase-like 1, transcript variant X5*
XM_019825689.2	*septin 7, transcript variant X7*
XM_019829713.2	*tubulin gamma complex associated protein 3, transcript variant X3*
XM_019832464.2	*EP300 interacting inhibitor of differentiation 1*
XM_019836585.2	*myocyte enhancer factor 2C, transcript variant X18*
XM_019836980.2	*Fas associated factor 1, transcript variant X1*
XM_019837265.1	*zinc finger ZZ-type containing 3, transcript variant X2*
XM_023238977.1	*pleckstrin homology-like domain family A member 2*
XM_023239126.1	*cell adhesion molecule 1, transcript variant X3*
XM_023240047.1	*calpain 1, transcript variant X1*
XM_023241058.1	*ornithine aminotransferase, transcript variant X2*
XM_023241314.1	*dynein axonemal heavy chain 10*
XM_023241586.1	*neurofibromin 2, transcript variant X2*
XM_023241792.1	*elastin microfibril interfacer 2, transcript variant X2*
XM_023241843.1	*SS18, nBAF chromatin remodeling complex subunit, transcript variant X3*
XM_023241980.1	*SMAD family member 7, transcript variant X2*
XM_023242133.1	*nuclear factor of activated T-cells 1, transcript variant X6*
XM_023242558.1	*thioredoxin*
XM_023242850.1	*cold inducible RNA binding protein, transcript variant X3*
XM_023243143.1	*transcription factor 3, transcript variant X8*
XM_023243448.1	*lysine demethylase 6B, transcript variant X7*
XM_023244057.1	*methyltransferase-like 23, transcript variant X2*
XM_023244808.1	*TATA-box binding protein associated factor 15, transcript variant X3*
XM_023245178.1	*POU class 2 homeobox 2, transcript variant X8*
XM_023246400.1	*mitogen-activated protein kinase 8 interacting protein 3, transcript variant X16*
XM_023247978.1	*lamin A/C*
XM_023248580.1	*family with sequence similarity 49 member B, transcript variant X6*
XM_023249097.1	*P2Y receptor family member 8, transcript variant X2*
XM_023249251.1	*dedicator of cytokinesis 11, transcript variant X1*
XM_023251817.1	*thymosin beta 10, transcript variant X2*
XM_023252054.1	*intersectin 2, transcript variant X7*
XM_023252112.1	*ornithine decarboxylase 1, transcript variant X2*
XM_023253670.1	*CD83 molecule, transcript variant X1*
XM_023253671.1	*CD83 molecule, transcript variant X2*
XM_023253685.1	*human immunodeficiency virus type I enhancer binding protein 1*
XM_023254138.1	*CD109 molecule, transcript variant X1*
XM_023254166.1	*synaptotagmin binding cytoplasmic RNA interacting protein, transcript variant X4*
XM_023255110.1	*transducing-like enhancer of split 3, transcript variant X7*
XM_023255350.1	*mitogen-activated protein kinase 6, transcript variant X2*
XM_023255486.1	*regulator of microtubule dynamics 3, transcript variant X3*
XM_023255629.1	*HECT domain E3 ubiquitin protein ligase 1, transcript variant X5*
XM_023256852.1	*cancer susceptibility 1, transcript variant X7*
XM_023256920.1	*dynamin-1-like, transcript variant X6*
XM_023257772.1	*adaptor-related protein complex 3 beta 1 subunit, transcript variant X1*
XM_023258773.1	*ubiquitin specific peptidase 1, transcript variant X3*
XM_023258859.1	*G protein subunit gamma 5*
XM_023260612.1	*nuclear receptor subfamily 1 group D member 2*
XM_023260684.1	*trafficking kinesin protein 1, transcript variant X8*
XR_002736856.1	*tetraspanin 14, transcript variant X2*
XR_002737850.1	*uncharacterized LOC111557626, transcript variant X1*
XR_002740181.1	*Eukaryotic 18S ribosomal RNA*
XR_890054.3	*heterogeneous nuclear ribonucleoprotein A2/B1, transcript variant X6*

**Table A2:** Genes differentially upregulated in all macrophages 17 h after infection with FIPV.

Gene Identifier	Gene Name
XM_003981024.5	*splicing regulatory glutamic acid and lysine rich protein 1, transcript variant X1*
XM_003981036.5	*cyclin B1*
XM_003981430.5	*3-hydroxy-3-methylglutaryl-CoA synthase 1, transcript variant X2*
XM_003981592.4	*centrosomal protein 72, transcript variant X2*
XM_003982742.5	*lanosterol 14-alpha demethylase, transcript variant X1*
XM_003982984.3	*aminoadipate-semialdehyde synthase, transcript variant X1*
XM_003984244.4	*cytoskeleton associated protein-2-like, transcript variant X1*
XM_003984844.5	*farnesyl-diphosphate farnesyltransferase 1, transcript variant X2*
XM_003984882.5	*methylsterol monooxygenase 1, transcript variant X1*
XM_003985082.5	*ELOVL fatty acid elongase 6, transcript variant X1*
XM_003986073.4	*FYVE, RhoGEF and PH domain containing 2, transcript variant X1*
XM_003987096.4	*cyclin B2, transcript variant X2*
XM_003988112.5	*optineurin, transcript variant X7*
XM_003988333.5	*cell division cycle associated 3, transcript variant X1*
XM_003988595.5	*Rap guanine nucleotide exchange factor 3, transcript variant X1*
XM_003988664.3	*trophinin associated protein*
XM_003990222.5	*zinc finger RANBP2-type containing 2, transcript variant X1*
XM_003992405.5	*myelin protein zero like-3, transcript variant X1*
XM_003993771.3	*7-dehydrocholesterol reductase, transcript variant X1*
XM_003995047.4	*NDC80, kinetochore complex component*
XM_003995401.5	*transmembrane protein 2, transcript variant X2*
XM_003995673.4	*Rho guanine nucleotide exchange factor 39*
XM_003996487.4	*sperm associated antigen 5, transcript variant X2*
XM_003996771.5	*SH3 and cysteine rich domain 2, transcript variant X1*
XM_003998762.5	*Polo-like kinase 1*
XM_003999323.5	*NIMA related kinase 2*
XM_003999347.5	*abnormal spindle microtubule assembly, transcript variant X2*
XM_004000594.4	*kinesin family member 4A*
XM_004000604.5	*mediator complex subunit 12*
XM_006927499.4	*fms-related tyrosine kinase 4, transcript variant X2*
XM_006928617.3	*phosphodiesterase 4C, transcript variant X3*
XM_006929641.4	*aurora kinase A*
XM_006932356.4	*Nei-like DNA glycosylase 1, transcript variant X1*
XM_006934172.3	*G2 and S-phase expressed 1, transcript variant X1*
XM_006934388.4	*zinc finger and BTB domain containing 40, transcript variant X2*
XM_006935662.3	*nuclear body protein SP140, transcript variant X7*
XM_006935975.4	*ribosomal oxygenase 2, transcript variant X1*
XM_006936407.2	*DNA topoisomerase II binding protein 1, transcript variant X3*
XM_006937682.4	*Ras association domain family member 7, transcript variant X2*
XM_006938581.4	*LIF, interleukin 6 family cytokine, transcript variant X2*
XM_006939451.4	*NIMA related kinase 6, transcript variant X2*
XM_006939718.4	*KIAA0753 ortholog, transcript variant X7*
XM_006943087.3	*farnesyl diphosphate synthase, transcript variant X3*
XM_011281000.3	*VPS54, GARP complex subunit, transcript variant X1*
XM_011281524.3	*fibroblast growth factor receptor 1, transcript variant X6*
XM_011283442.3	*tyrosyl-DNA phosphodiesterase 1, transcript variant X1*
XM_011284205.3	*cytoskeleton associated protein 2*
XM_011284366.2	*DENN domain containing 6B, transcript variant X2*
XM_011287426.3	*centrosomal protein 55, transcript variant X3*
XM_011287724.3	*checkpoint kinase 2, transcript variant X2*
XM_011288029.3	*regulation of nuclear pre-mRNA domain containing 1A, transcript variant X1*
XM_011288421.2	*syntaxin binding protein 1, transcript variant X2*
XM_011289261.3	*sclerostin*
XM_011289687.3	*carnitine palmitoyltransferase 1C, transcript variant X1*
XM_011290536.3	*F-box and leucine rich repeat protein 19, transcript variant X1*
XM_011290681.3	*brain-specific serine protease 4, transcript variant X4*
XM_011291526.3	*kinesin family member C2, transcript variant X2*
XM_019812837.2	*7-dehydrocholesterol reductase, transcript variant X3*
XM_019813506.2	*poly(ADP-ribose) glycohydrolase, transcript variant X3*
XM_019816169.1	*talin 1, transcript variant X2*
XM_019817080.2	*discs large MAGUK scaffold protein 4, transcript variant X9*
XM_019817127.2	*aurora kinase B, transcript variant X3*
XM_019817247.2	*angiopoietin-like 6, transcript variant X2*
XM_019817546.2	*synergin gamma, transcript variant X8*
XM_019817554.1	*acetyl-CoA carboxylase alpha, transcript variant X5*
XM_019817596.2	*proline rich 11, transcript variant X3*
XM_019817950.2	*ETS variant 4, transcript variant X5*
XM_019819207.2	*interferon regulatory factor 3, transcript variant X3*
XM_019820028.2	*fucokinase, transcript variant X1*
XM_019821899.1	*complement C3d receptor 2, transcript variant X3*
XM_019821902.1	*complement C3d receptor 2, transcript variant X6*
XM_019822306.2	*upstream transcription factor 1, transcript variant X4*
XM_019824070.1	*centromere protein I*
XM_019826681.2	*TPX2, microtubule nucleation factor, transcript variant X7*
XM_019829073.2	*alpha kinase 1, transcript variant X4*
XM_019830601.2	*peroxisomal biogenesis factor 6, transcript variant X3*
XM_019830890.2	*TTK protein kinase, transcript variant X6*
XM_019831446.2	*acetyl-CoA acetyltransferase 2*
XM_019834535.2	*apolipoprotein L domain containing 1, transcript variant X1*
XM_019834543.2	*apolipoprotein L domain containing 1, transcript variant X8*
XM_019835370.2	*apoptotic peptidase activating factor 1, transcript variant X1*
XM_019836058.2	*nephrocystin 4, transcript variant X2*
XM_019839257.2	*lanosterol synthase, transcript variant X3*
XM_023239847.1	*telomerase reverse transcriptase, transcript variant X11*
XM_023239925.1	*fatty acid desaturase 2, transcript variant X1*
XM_023240698.1	*chondroitin sulfate N-acetylgalactosaminyltransferase 2, transcript variant X8*
XM_023241280.1	*acetoacetyl-CoA synthetase, transcript variant X2*
XM_023241432.1	*HECT domain E3 ubiquitin protein ligase 4, transcript variant X2*
XM_023242979.1	*vav guanine nucleotide exchange factor 2, transcript variant X7*
XM_023243604.1	*zinc finger and BTB domain containing 4, transcript variant X3*
XM_023243877.1	*myosin XVIIIA, transcript variant X20*
XM_023244268.1	*ring finger protein 213, transcript variant X5*
XM_023244594.1	*centrobin, centriole duplication and spindle assembly protein, transcript variant X3*
XM_023244664.1	*acetyl-CoA carboxylase alpha, transcript variant X9*
XM_023244901.1	*mevalonate diphosphate decarboxylase, transcript variant X1*
XM_023245382.1	*dynamin 2, transcript variant X5*
XM_023245498.1	*dedicator of cytokinesis 6*
XM_023245844.1	*lipase E, hormone sensitive type, transcript variant X4*
XM_023245914.1	*DM1 locus, WD repeat containing, transcript variant X1*
XM_023246136.1	*LSM14A, mRNA processing body assembly factor, transcript variant X1*
XM_023246494.1	*cyclin F*
XM_023247155.1	*chromosome E3 C16orf62 homolog, transcript variant X4*
XM_023248638.1	*protein tyrosine kinase 2, transcript variant X19*
XM_023249981.1	*tRNA nucleotidyl transferase 1, transcript variant X3*
XM_023250648.1	*EPH receptor B6, transcript variant X1*
XM_023250847.1	*protein tyrosine phosphatase, receptor type S, transcript variant X10*
XM_023250896.1	*insulin induced gene 1*
XM_023252016.1	*elastin microfibril interfacer 1*
XM_023252392.1	*pericentriolar material 1, transcript variant X15*
XM_023252891.1	*centromere protein E, transcript variant X7*
XM_023252992.1	*heterogeneous nuclear ribonucleoprotein D, transcript variant X6*
XM_023253738.1	*discoidin domain receptor tyrosine kinase 1, transcript variant X1*
XM_023253977.1	*ubiquitin protein ligase E3 component n-recognin 2, transcript variant X3*
XM_023254254.1	*cyclin dependent kinase 19, transcript variant X4*
XM_023254265.1	*FYN proto-oncogene, Src family tyrosine kinase, transcript variant X1*
XM_023255117.1	*kinesin family member 23, transcript variant X2*
XM_023255227.1	*HECT and RLD domain containing E3 ubiquitin protein ligase family member 1, transcript variant X3*
XM_023255500.1	*BUB1 mitotic checkpoint serine/threonine kinase B*
XM_023255706.1	*DLG associated protein 5, transcript variant X1*
XM_023256274.1	*isopentenyl-diphosphate delta-isomerase 1, transcript variant X2*
XM_023256524.1	*cullin 2, transcript variant X1*
XM_023257105.1	*integrin subunit beta 7, transcript variant X5*
XM_023257126.1	*diacylglycerol kinase alpha, transcript variant X2*
XM_023257348.1	*thymopoietin, transcript variant X4*
XM_023257700.1	*casein kinase I, transcript variant X4*
XM_023257872.1	*Holliday junction recognition protein*
XM_023259342.1	*plakophilin 4, transcript variant X6*
XM_023259713.1	*Obscurin-like 1, transcript variant X6*
XM_023260160.1	*cytoplasmic FMR1 interacting protein 2, transcript variant X2*
XR_002150997.2	*uncharacterized LOC109495548*
XR_002160083.2	*MFNG O-fucosylpeptide 3-beta-N-acetylglucosaminyltransferase, transcript variant X2*
XR_002737671.1	*phosphatidylinositol-4-phosphate 5-kinase-like 1, transcript variant X2*
